# A new species of the cave-fish genus *Lucifuga* (Ophidiiformes, Bythitidae), from eastern Cuba

**DOI:** 10.3897/zookeys.946.51373

**Published:** 2020-07-06

**Authors:** Damir Hernández, Peter Rask Møller, Didier Casane, Erik García-Machado

**Affiliations:** 1 Centro de Investigaciones Marinas, Universidad de La Habana, Calle 16, No. 114 entre 1ra y 3ra, Miramar, Playa, Ciudad Habana 11300, Cuba Centro de Investigaciones Marinas, Universidad de La Habana Havana Cuba; 2 Natural History Museum of Denmark, University of Copenhagen, Universitetsparken 15, DK-2100 Copenhagen Ø, Denmark Natural History Museum of Denmark Copenhagen Denmark; 3 Université Paris-Saclay, CNRS, IRD, UMR Évolution, Génomes, Comportement et Écologie, 91198, Gif-sur-Yvette, France Université Paris-Saclay Gif-sur-Yvette France; 4 Université Paris Diderot, Sorbonne Paris Cité, 5 rue Thomas-Mann, 75205 Paris, France Université Paris Diderot, Sorbonne Paris France; 5 Institut de Biologie Intégrative et des Systèmes, Université Laval, Québec, QC, G1V 0A6, Canada Institut de Biologie Intégrative et des Systèmes, Université Laval Québec Canada

**Keywords:** Anchialine caves, Gibara, Holguin, speleology, taxonomy, viviparous brotulas

## Abstract

Recently, a barcoding study and a molecular phylogenetic analysis of the Cuban species of the cave-fish genus *Lucifuga* Poey, 1858 revealed the existence of different evolutionary lineages that were previously unknown or passed unnoticed by morphological scrutiny (i.e., cryptic candidate species). In the present study, *Lucifuga gibarensis* is described as a new species restricted to anchialine caves in the northeastern karst region of the main island. The species was earlier described as a variety of *Lucifuga dentata*, but since the name was introduced as a variety after 1960, it is deemed to be infrasubspecific and unavailable according to the International Code of Zoological Nomenclature Art. 15.2. The new species differs from *L. dentata* by pigmented eyes vs. eyes absent and lack of palatine teeth vs. present. *Lucifuga gibarensis* seems to be most similar to the Bahamian species *L. lucayana* by showing pigmented eyes, 13 or 14 precaudal vertebrae and ten caudal fin rays. However, differs from it by a larger size of the pigmented eye (1.1–1.9 vs. 0.9–1.0% SL) and number of posterior lateral line neuromasts (30–33 vs. 34–35).

## Introduction

*Lucifuga* Poey, 1858 is a conspicuous genus of obligate cave-dwelling fishes, currently recognised with six species distributed in Cuba and Bahamas (Nielsen et al. 1999; Møller et al. 2006, 2016; see comparative material). Another nominal species, *Lucifuga inopinata* Cohen and McCosker, 1998, from off Galapagos Archipelago belongs to another, yet undescribed, genus (Møller unpublished data).

Because of the characteristics of the habitats of *Lucifuga* species (caves, sinkholes and crevices) and the morphological modifications that they show in the evolutionary adaptations to the environment, the genus represents an iconic part of the fish fauna in Cuba. The scientific interest in these fishes, however, has been sporadic. Since the description of the genus and the two first Cuban species by Felipe Poey (1858), the studies dealing with the genus are very few and have mainly been dedicated to the discussions of morphological characters of taxonomic interest for the genus and species and the descriptions of new species (Gill 1863; Nalbant 1981; Díaz-Pérez et al. 1987a, 1987b; Díaz-Pérez 1988); some aspects of feeding and reproductive system (Lane 1903; Eigenmann 1909; Thinès and Piquemal 1978; García-Debrás and Pérez 1999) and two studies that constituted the first approximation to the evolutionary relationships of the group based on a comparison of several morphological characters of the three species known at that time (Vergara 1980, 1981).

Møller et al. (2006) found evidence for all Cuban and all Bahamian species representing two separate evolutionary lineages, but recently García-Machado et al. (2011) made a phylogenetic analysis of the Cuban species using mitochondrial and nuclear genes finding several new evolutionary lineages not identified previously by morphological analyses. It was also indicated that the separation in Cuban and Bahamian species as suggested by Møller et al. (2006) is no longer correct, since some of the new Cuban species are more closely related to Bahamian species than to other Cuban species. Their results also questioned the specific status of *Lucifuga teresinarum* Diaz, 1988, showing no difference to *L. subterranea* Poey, 1858 (see also Lara et al. 2010).

A controversial taxon has been Lucifuga dentatus var. holguinensis Díaz-Pérez, Nieto and Abio, 1987 from the Holguin province in eastern Cuba. It was suggested as a valid species name by Proudlove (2019), but the name has now been decided to be infrasubspecific and unavailable according to ICZN Art. 15.2, since it was introduced as a variety after 1960 (Fricke et al. 2019). In the present study, based on the molecular results of García-Machado et al. (2011) and from revisiting the morphological characters recently used to define species in the genus (Møller et al. 2006), we present a new formal description of the species as *Lucifuga gibarensis* sp. nov.

## Materials and methods

**Figure 1.**
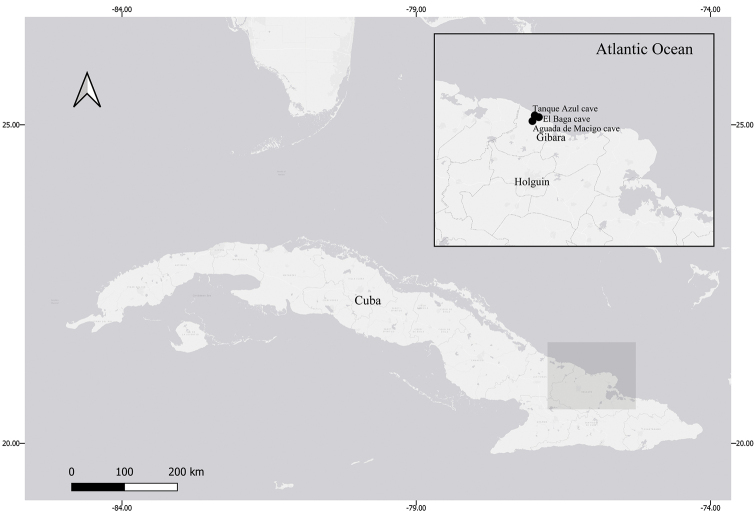
Sample sites of *Lucifuga gibarensis* sp. nov. in eastern Cuba.

The morphological study of the Cuban *Lucifuga* species was based on the analysis of 214 individuals sampled from several localities covering most of its known distribution areas (Fig. 1). Nine morphometric measurements were taken using a Vernier calliper (precision 0.05 mm) and eleven meristic counts (e.g., fin ray numbers, scales, etc.) were carried out using a Novel stereomicroscope (magnification 40 x maximum) and/ or radiographs. All morphometric measurements were weighted according to the standard length (SL) to avoid allometric effects. The number of vertebrae was counted using X-ray radiographs.

Abbreviations include:

**ANSP**Academy of Natural Sciences of Drexel University, Philadelphia, Pennsylvania, U.S.A;

**FMNH** Division of Fishes, Department of Zoology, Field Museum of Natural History, Chicago, Illinois, U.S.A.;

**MCZ** Museum of Comparative Zoology, Harvard University, Ichthyology Department, Cambridge, Massachusetts, U.S.A;

**MFP**Felipe Poey Museum of Havana University, Cuba;

**UMMZ**University of Michigan Museum of Zoology, Ann Arbor, Michigan, U.S.A.;

**ZMB**Museum für Naturkunde, Leibniz-Institut für Evolutions- und Biodiversitätsforschung, Berlin, Germany;

**ZMUC**Natural History Museum of Denmark, University of Copenhagen, Denmark.

## Taxomomy

**Family Bythitidae**

### Genus Lucifuga

Taxon classificationAnimaliaOphidiiformesBythitidae

Poey, 1858

E967C091-D7DA-5888-AB98-4FBF8C8621A4

#### Type species.

*Lucifuga subterranea* Poey, 1858 by subsequent designation of Jordan and Evermann, 1896, type locality: El Cajio cave, but not precisely stated for *L. subterranea*, which was referred originally from caves of San Antonio, middle-south Havana province, Cuba.

#### Diagnosis.

Body moderately elongated and compressed mainly from the abdomen to the caudal end. Snout with two nostrils: anterior nostril tube-shape and smaller, placed near to the upper lip; posterior nostril is a larger hole, placed ca. midway between snout and eyes cavity. The mouth is subterminal with the lower jaw only slightly shorter than the upper. Opercular spines absent. Seven branchiostegal rays.

The entire body is covered with small, rounded cycloid scales; fins naked except for scales on pectoral fin basis. Predorsal area and operculum scaled. Branchiostegal membranes, entire underside of the head, snout, interorbital areas and entire course of the cavernous cephalic system are naked. Origin of dorsal fin approximately above the tip of pectoral fins. Pelvic fin is subjugular with a single ray reaching ca. 1/3 to halfway to the anus. Pectoral fin behind the operculum, peduncle short and narrow. Lateral line with two series of sensory neuromasts: upper and anterior series extends from the head to a point ca. midway between dorsal and anal fin origins; and lower and posterior series extends from a point under and slightly in advance of the end of the upper series to the mid side from the caudal base.

There are three symmetric sensory canal series on each side of the head: supraorbital series with three pores (two anterior and one posterior): the anteriormost is at the snout rim, the second open between and above the nasal openings, and the posterior single pore is at the end of the lateral canal above the operculum. The infraorbital series with six pores (three anterior and three posterior): first pore is slightly below the anterior nasal opening, the other five pores (two anterior and three posterior) are along the edge of infraorbital rim. Finally, the mandibular series with six pores (three anterior and three posterior). The first pore is in the fold of skin between the lip and canal series, the second is at the side of the jaw tip on the lower lip, the third is at the anterior end of the mandibular series, the fourth to sixth posterior pores open ventrally along the mandibular series. There is also a large preopercular pore. Teeth are present on the premaxillae, dentaries and vomer; but are present or absent in palatines.

Sexual dimorphism. The male copulatory organ is completely integrated into a fleshy genital hood which projects posteriorly beyond the anus, the lateral end of the hood could be from broad to conical. A fleshy small conical papillae project from the middle of the distal margin of the hood and is enclosed by lateral earlike lobes. Penis is placed underneath the hood.

### Lucifuga gibarensis sp. nov.

Taxon classificationAnimaliaOphidiiformesBythitidae

C405C4C7-5BE3-52CC-B40A-F6795BFF3F8F

http://zoobank.org/4D8B142C-4CAE-49CB-B93D-42FA40B266A9

**Figure 2.**
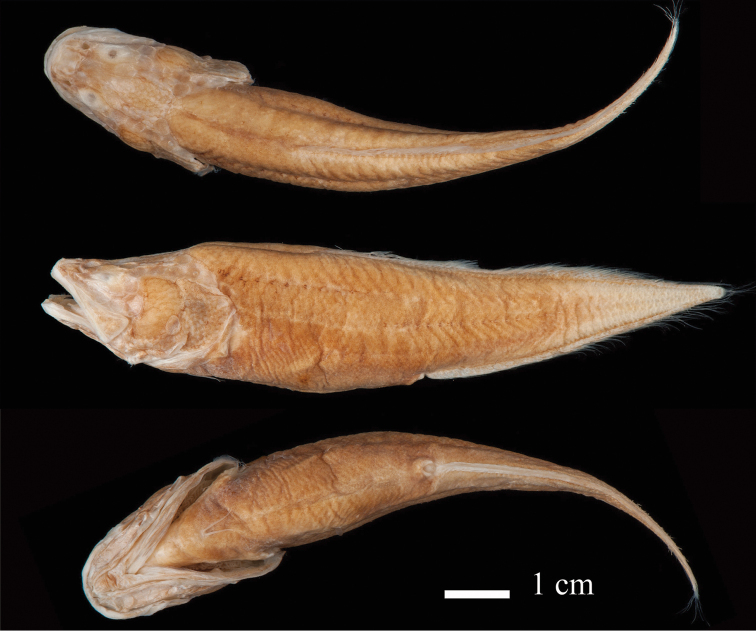
*Lucifuga gibarensis* sp. nov. Holotype, MFP 18.000420, 89.3 mm SL, female, Aguada de Macigo cave, Gibara municipality, Northern Holguin province, Cuba.

**Figure 3.**
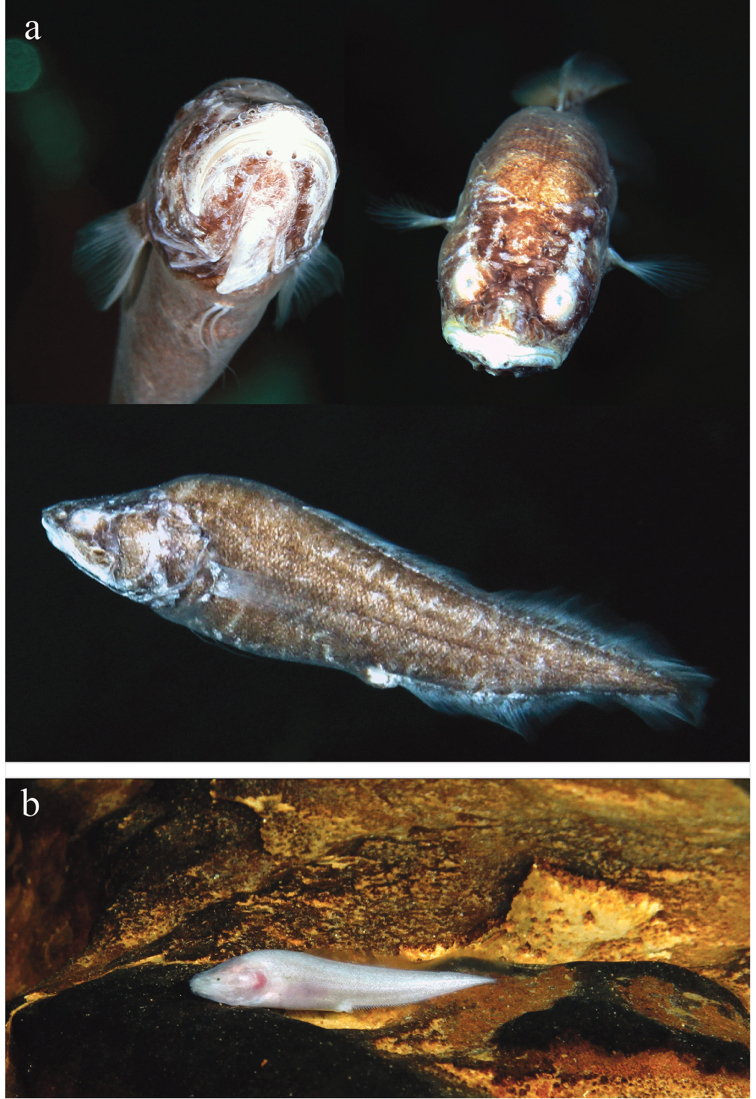
*Lucifuga gibarensis* sp. nov. in Cueva El Baga, Gibara municipality, northern Holguin province, Cuba, **a** unsampled specimen, 27 November 2014 **b**ZMUC P771732, 45.0 mm SL, male, photo taken immediately prior to collection.

Figures 1 , 2 , 3 ; Tables 1 , 2 Common name: Gibara cave brotula (English)

Lucifuga dentatus variety holguinensis Díaz-Pérez et al., 1987b: 44.
Lucifuga dentatus var. holguinensis Hernández 2005: 15; García-Machado et al. 2011: 471.
Lucifuga holguinensis Proudlove 2019.

#### Holotype.

MFP 18.000420, 89.3 mm SL, female, Aguada de Macigo cave, ca. 21°09'42"N, 76°14'55"W, near Gibara municipality, Northern Holguin province, Cuba, collected by Eduardo Nieto, in 1986, designated as *Lucifuga dentatus* variety *holguinensis* by Díaz-Pérez et al. (1987b).

#### Paratypes.

MFP 18.000399, 69.3 mm SL, male, Tanque Azul cave, ca. 21°12'6"N, 76°13'59"W, near Gibara municipality, Northern Holguin province, Cuba, collected by Alfredo García-Debrás, 2 June 1997; MFP 18.000278, 89.2 mm SL, male, Aguada de Macigo cave, Gibara municipality, Northern Holguin province, Cuba, collected by Arturo Rojas, 21 November 2014; ZMUC P771732, 45.0 mm SL, male, Cueva El Baga, ca. 21°11'51"N, 76°14'3"W, near Gibara municipality, northern Holguin province, Cuba, collected by Katrine Worsaae and Peter Rask Møller, 27 November 2014.

#### Diagnosis.

Dorsal fin rays 72–90; anal fin rays 58–72; pectoral fin rays 15–17, caudal fin rays 10; palatine teeth absent; rakers on anterior gill arch 17–19 (long gill-rakers 3); occiput and area between lateral canal and preopercular canal scaled; diameter of pigmented eyes 1.1–1.9% SL; total vertebrae 50–53.

#### Description.

Meristic and morphometric characters are given in Tables 1, 2. Body moderately elevated behind the head, with a slight depression in the interorbital region (Figs 2, 3). Eyes pigmented (similar to the condition present in *L. spelaeotes* and *L. lucayana* (Møller et al. 2006)). Anterior gill arch with three elongate rakers and 14–16 low dentigerous pads. The areas between lateral canal and preopercular canal, and the occiput are scaled (Fig. 2). Caudal fin free (not fused with dorsal and anal fins). In the lateral line series of sensory neuromasts, the upper and anterior count with 13–15, the lower and posterior with 30–35. Teeth are present on the premaxillae (5–7 rows), dentaries (6 or 7 rows) and vomer (2 or 3 rows in two separate patches). Palatines without teeth.

**Table 1.** Morphometric and meristic characters of *Lucifuga* spp. (HT: holotype; PT: paratype; ST: syntype).

	*L. gibarensis* sp. nov.		*L. dentata*		*L. simile*		*L. subterranea*	*L. lucayana*		*L. spelaeotes*	
	HT	HT and 3 PT	ST, MCZ 32329	2 ST and 126 nontypes	22 nontypes	HT	HT and 42 nontypes	HT	HT and 5 PTs	HT	HT, PT and 40 nontypes
		Mean and range		Mean and range	Mean and range		Mean and range		Mean and range		Mean and range
SL (mm)	89.3	73.2 (45.0–89.3)	85.0	91.0 (45–124)	74.5 (57.5–103)	69.0	66.1 (39.7–89.5)	99.0	74.3 (44–99)	110	106.3 (42–166)
Morphometric characters (% SL)											
Head length	26.0	27.5 (26.0–28.4)	28.5	26.7 (18.4–31.5)	24.0 (17.1–31.2)	28.7	26.3 (18.8–28.9)	28.8	28.4 (27.1–29.3)	29.1	28.8 (26.2–31.3)
Jaw length	13.4	14.5 (13.4–15.3)	14.9	14.3 (11.7–20.2)	14.7 (11.2–17.0)	13.3	12.0 (9.6–14.4)	14.9	14.2 (13.2–14.9)	16.2	14.7 (12.4–16.8)
Maximum height	20.4	19.1 (17.8–20.4)	–	19.8 (13.1–24.5)	20.8 (14.4–26.0)	–	16.9 (13.2–21.7)	–	–	–	–
Diameter of pigmented eye	1.5	1.4 (1.1–1.9)	0.0	0.02 (0.0–0.2)	0.0	0.0	0.1 (0.0–0.3)	1.0	1.0 (0.9–1.0)	1.3	1.3 (0.7–1.8)
Predorsal length	39.1	37.6 (35.9–39.1)	39.6	40.1 (31.5–50.7)	40.2 (32.9–45.3)	40.8	39.9 (36.3–44.0)	37.0	36.4 (35.5–37.0)	39.9	38.1 (34.7–41.1)
Preanal length	58.2	55.7 (52.6–58.3)	53.7	54.6 (50.0–65.8)	54.7 (44.0–61.3)	55.4	54.0 (48.2–59.4)	55.6	55.0 (51.7–57.6)	54.1	54.2 (48.2–60.5)
Pectoral fin length	11.2	13.7 (11.2–15.3)	11.4	10.6 (7.5–15.7)	10.2 (8.0–12.5)	10.3	8.9 (7.8–12.0)	13.3	12.6 (11.3–13.3)	12.5	12.7 (11.1–14.4)
Base of pelvic fin to anal fin origin	35.9	35.6 (32.2–38.7)	31.5	31.5 (23.0–38.1)	28.9 (20.3–33.8)	31.6	31.6 (26.9–35.5)	34.9	33.7 (29.4–36.9)	31.3	31.2 (27.0–36.9)
Dorsal fin origin to anal fin origin	17.8	18.2 (17.8–18.9)	–	14.3 (9.4–18.4)	14.6 (12.3–16.9)	–	14.2 (9.3–18.8)	–	–	–	–
Meristic characters											
Dorsal fin rays	72	82.3 (72–90)	–	90.5 (82–102)	72.9 (67–80)	87	82.8 (70–87)	91	89.2 (84–91)	92	97.5 (86–109)
Anal fin rays	58	65.5 (58–72)	–	72.4 (66–80)	58.4 (54–70)	71	64.2 (53–70)	67	66.2 (63–69)	71	73.8 (66–82)
Caudal fin rays	10	10	–	8	8	8	8	10	10	10	10
Pectoral fin rays	16	16.0 (15–17)	15	16.1 (15–17)	14.4 (13–17)	13	11.9 (10–13)	17	17.2 (17–18)	18	18.5 (17–20)
Precaudal vertebrae	14	13.5 (13–14)	–	11.1(11–12)	11	11	11.3 (11–12)	13	12.8 (12–13)	13	13.2 (13–14)
Caudal vertebrae	36	36.5 (36–37)	–	35.9(34–37)	34–35	37	35.8 (34–37)	39	38.6 (37–39)	39	39.5 (38–42)
Total vertebrae	50	51.0 (50–53)	–	47.0(46–48)	46	48	46.9 (46–48)	52	51.3 (50–52)	52	52.7 (51–55)
Rakers on anterior gill arch	18	18.0 (17–19)	20	18.4 (15–22)	17.6 (15–20)	16	14.2 (12–17)	16	15.3 (13–17)	21	18.8 (15–23)
Premaxillary teeth rows	5	6.0 (5–7)	6	4.3 (3–6)	3.9 (3–5)	8	4.0 (3–8)	7	6.0 (5–7)	8	6.8 (4–10)
Palatine teeth rows	0	0	3	2.1 (1–4)	2.2 (1–4)	0	0	0	0	5	3.7 (1–7)
Lateral line neuromasts	15/ 30	13–15/ 30–33	–	12–18/ 22–33	12–15/ 24–27	–	12–19/ 26–33	13/ 35	12–13/34–35	14/38	12–19/30–47
Occiput squamation	Yes	Yes	–	No 79%/Yes 21%	No		Yes		Yes		Yes

#### Coloration.

Uniformly brown or light brown, with lighter fins and naked parts on the head. Nevertheless, one juvenile specimen (ZMUC P771732) was very pale, but still with tiny dark pigment dots (Fig. 3b).

#### Distribution and habitat.

*Lucifuga gibarensis* shows a very restricted known distribution, in a lithographically isolate karst patch of caves at the north of Gibara municipality, Holguín province, without any overlap with other Cuban species of the genus (Fig. 1; García-Machado et al. 2011; Hernández et al. 2016). It is ca. 800 km away from the nearest *L. dentata*, *L. subterranea* and *L. simile* distribution areas. The distance to the Bahamian species on Little Bahama Bank (*L. lucayana*) and Great Bahama Bank (*L. spelaeotes*) is ca. 650 km and 240 km, respectively. The location area is composed by three caves (Aguada de Macigo, Tanque Azul and Cueva El Baga) located near to the shore ca. 3–15 km from each other (Corella et al. 2000, Dietz 2015). The Aguada de Macigo cave is the type-locality with an emergent large doline, ca. 22 m deep and salinity of 16 ppt. According to Díaz-Pérez et al. (1987b), the individual designated as holotype was caught at 12 m depth.

**Table 2.** Frequency of meristic characters in *Lucifuga* spp.

**Number of dorsal fin rays**																																								
	**6 7**	**6 9**	**7 0**	**7 1**	**7 2**	**7 4**	**7 5**	**7 6**	**7 7**	**7 8**	**7 9**	**8 0**	**8 1**	**8 2**	**8 3**	**8 4**	**8 5**	**8 6**	**8 7**	**8 8**	**8 9**	**9 0**	**9 1**	**9 2**	**9 3**	**9 4**	**9 5**	**9 6**	**9 7**	**9 8**	**9 9**	**1 0 0**	**1 0 1**	**1 0 2**	**1 0 3**	**1 0 4**	**1 0 5**	**1 0 6**	**1 0 9**	**N**
*L. dentata*														1	3	1	1	2	6	5	5	5	5	6	5	7	3	2	1					1						61
*L. gibarensis* sp. nov.					1										1	1						1																		4
*L. simile*	1	2	2	2	1	2	2	3	2			1																												18
*L. subterranea*			1					1	5	4	5	7	3	3	7	2	2	1																						41
*L. lucayana*																1					1		4																	6
*L. spelaeotes*																		1			1		2	2	1	6	2	3	2	5	2	6	1	2	1	1	2	1	1	42
**Number of anal fin rays**																																								
	**5 3**	**5 4**	**5 5**	**5 6**	**5 7**	**5 8**	**6 0**	**6 1**	**6 2**	**6 3**	**6 4**	**6 5**	**6 6**	**6 7**	**6 8**	**6 9**	**7 0**	**7 1**	**7 2**	**7 3**	**7 4**	**7 5**	**7 6**	**7 7**	**7 8**	**7 9**	**8 0**	**8 1**	**8 2**	**N**										
*L. dentata*													4	3	3	4	5	6	6	7	4	4	4	3	3	2	3			61										
*L. gibarensis* sp. nov.						1						1		1					1											4										
*L. simile*		2	2	3	3	3	1	1		1	1						1													18										
*L. subterranea*	1						2	6		7	8	4	6	3	3		1													41										
*L. lucayana*										1			2	1		1														5										
*L. spelaeotes*													1	1	1	1	2	7	6	4	4	2	2	2	2	4	1	1	1	42										
**Number of pectoral fin rays**																																								
	**1 0**	**1 1**	**1 2**		**1 3**		**1 4**	**1 5**		**1 6**		**1 7**		**1 8**		**1 9**		**2 0**	**N**																					
*L. dentata*								22		48		24							94																					
*L. gibarensis* sp. nov.								1		1		1							3																					
*L. simile*					5		8	4		3		1							21																					
*L. subterranea*	3	7	25		10														45																					
*L. lucayana*												5		1					6																					
*L. spelaeotes*												2		18		20		2	42																					
**Number of posterior lateral line neuromasts**																																								
	**2 0**	**2 2**	**2 3**	**2 4**	**2 5**	**2 6**	**2 7**	**2 8**	**2 9**	**3 0**	**3 1**	**3 2**	**3 3**	**3 4**	**3 5**	**3 6**	**3 7**	**3 8**	**3 9**	**4 0**	**4 1**	**4 2**	**4 3**	**4 4**	**4 5**	**4 6**	**4 7**	**N**												
*L. dentata*		1	1	1	3	5	7	1	4	3			1															27												
*L. gibarensis* sp. nov.										2			1															3												
*L. simile*				2		2	2																					6												
*L. subterranea*							1	7	4	8	2	4	3															29												
*L. lucayana*														1	1													2												
*L. spelaeotes*										1					2	1	1	2	5	6	1	2				1	1	23												
**Number of rakers on anterior gill arch**																																								
	**1 2**	**1 3**	**1 4**		**1 5**	**1 6**	**1 7**	**1 8**		**1 9**		**2 0**	**2 1**	**2 2**	**2 3**	**N**																								
*L. dentata*					2	3	6	11		10		7	2	1		42																								
*L. gibarensis* sp. nov.							1	1		1						3																								
*L. simile*					1	1	3	2		2		1				10																								
*L. subterranea*	3	9	10		9	2	1									36																								
*L. lucayana*		1				1	1									3																								
*L. spelaeotes*					3	3	6	3		9		5	8	1	1	39																								

#### Etymology.

The specific epithet refers to the village of Gibara, where the three caves inhabited by this species are located. We do not follow variety epithet used by Díaz-Pérez et al. (1987b), since the *L. gibarensis* better describes the narrow distribution of the species near the village Gibara instead of the entire region Holguin.

#### Genetic distances.

Among Cuban species, García-Machado et al. (2011) have demonstrated that *L. gibarensis* [at that time as L. dentata var. holguinensis] is not phylogenetically close to *L. dentata* by showing a large mitochondrial DNA divergence of 30.5% (16.5% with cytochrome *b* gene) as well as several diagnostic nucleotide variations at nuclear genes. In contrast, *L. gibarensis* is phylogenetically closely related to other two lineages of undescribed species of *Lucifuga* from Cuba (named *Lucifuga* sp. 3 and *L.* sp. 4) (García-Machado et al. 2011). However, genetic distance to both Bahamian species is not yet known.

#### Comparisons.

Based on external appearance, *Lucifuga gibarensis* sp. nov. resembles the Cuban species *L. dentata* (from which it was designated as variety, see Díaz et al. 1987b) and *L. simile*. Nonetheless, it differs in several characters: e.g., number of caudal fin rays (10 vs. 8), diameter of the pigmented eyes (1.1–1.9 vs. 0.0–0.2% SL), lack of palatine teeth vs. present and scaled occiput vs. naked or weakly scaled occiput. It also differs in dorsal and anal fin rays mean number (fewer than *L. dentata* and more than *L. simile*) (Table 1).

*Lucifuga gibarensis* sp. nov. also resembles *L. subterranea* in the lack of palatine teeth and the scaled occiput, but it differs in the body moderately elevate behind the head vs. little elevated (see maximum height in Table 1), number of pectoral fin rays (15–17 vs. 10–13), number of caudal fin rays (10 vs. 8), the diameter of the pigmented eyes (1.1–1.9 vs. 0.0–0.3% SL) and in the number of rakers on the anterior gill arch 17–19 vs. 12–17 (Table 1).

Finally, *Lucifuga gibarensis* resembles both Bahamian species in the head profile, the number of caudal fin rays (10), the occiput scales (similar to *L. spelaeotes* and less scaled than *L. lucayana*) and in the presence of relatively large pigmented eyes (Table 1). With *L. lucayana* it also shares the lack of palatine teeth. It differs in the number of pectoral fin rays (15–17 vs. 17–18 in *L. lucayana* and 17–20 in *L. spelaeotes*); and diameter of pigmented eye is larger than in *L. lucayana* (1.1–1.9 vs. 0.9–1.0% SL).

#### Remarks.

It has been demonstrated that *L. gibarensis* is not phylogenetically close to *L. dentata*. The estimate of mtDNA genetic divergence between these two lineages is huge (P = 30.5%) and several diagnostic nucleotide changes at the intron 4 of calmoduline gene and intron 1 of the homeodomain EVX gene were described (García-Machado et al. 2011). Designation as a variety of *L. dentata*, was wrong as judgment, given the sharp differences observed at three major morphological characters: palatine teeth; number of caudal fin rays; and degree of pigmentation in the eyes. Particularly, the number of caudal fin rays (10) and pigmented eyes were realised in *L. spelaeotes* description (Cohen and Robins 1970), and recognised as diagnostic characters to distinguish the Cuban and Bahamian species at that time (Cohen and Robins 1970; Møller et al. 2006).

As a result of the present study, we describe a new species, *Lucifuga gibarensis*, which is supported by morphology and molecular phylogenetic analysis (García-Machado et al. 2011). We found unique diagnostic characters that distinguish this species from all the species described so far. Díaz-Pérez et al. (1987b) identified this *taxon* as a variety of *Lucifuga dentata* (L. dentata var. holguinensis), and recognised the presence of 10 caudal fin rays and pigmented eyes (characters distinguished by Cohen and Robins (1970) as important to separate *L. spelaeotes* from the two Cuban species known at that time), but underestimated the taxonomic relevance of these characters and avoid them. They also underrated the absence of palatine teeth vs. present in *L. dentata*, a useful taxonomic character to distinguish *Lucifuga* species (see Poey 1858; Møller et al. 2006). Furthermore, Møller et al. (2006) pointing out that the Bahamian species differing from all four Cuban species formerly known by having higher caudal fin rays number (10 vs. 8), larger pigmented eyes diameter (0.7–1.8 vs. 0.0–0.3% SL), higher vertebrae number (50–55 vs. 45–48), and higher pectoral fin rays number (17–20 vs. 10–17) supporting the hypothesis that Bahamas and Cuba are represented by two different evolutionary lineages (see also Vergara 1980, 1981). However, the new Cuban species *L. gibarensis*, shared a similar combination of these characters with Bahamian species apart from low number of pectoral fin rays in *L. gibarensis*. Based on these characters, our results do not support that lineages are confined to only one Archipelago. With the available knowledge, species with reduced or completely absence of eyes and 8 fin rays are only found in western Cuba; but species having pigmented eyes and 10 caudal fin rays are found in both archipelagos. Detailed phylogenetic studies including all Atlantic *Lucifuga* spp. will be crucial to clarify the phylogeographic relationships between the Cuban and Bahamian members of this genus.

### Identification key to species of *Lucifuga*

The current key is based on a small number of samples. Measures that overlapping in range were only used when it helps distinguishing between two species.

**Table:** 

1	Diameter of pigmented eyes 0.0–0.3% SL, caudal fin rays 8, number of vertebrae < 50	**2**
–	Diameter of pigmented eyes > 0.7% SL, caudal fin rays 10, number of vertebrae ≥ 50	**4**
2	Palatine teeth present, pectoral fin rays 13–17	**3**
–	Palatine teeth absent, pectoral fin rays 10–13	***L. subterranea***
3	Dorsal fin rays < 80	***L. simile***
–	Dorsal fin rays ≥ 80	***L. dentata***
4	Palatine teeth present, lateral occipital area naked	***L. spelaeotes***
–	Palatine teeth absent, lateral occipital area scaled	**5**
5	Pigmented eye diameter 0.9–1.0% SL, number of posterior lateral line neuromasts 34–35	***L. lucayana***
–	Pigmented eye diameter 1.1–1.9% SL, number of posterior lateral line neuromasts 30–33	***L. gibarensis* sp. nov.**

### Comparative material

### Lucifuga subterranea

Taxon classificationAnimaliaOphidiiformesBythitidae

Poey, 1858

E4F50EFB-6E61-5266-AFD5-CB57536877DF

#### Material examined.

(38 specimens: 18 females, 20 males).

***Holotype***: ZMB 6314, 69 mm SL, female, Cueva de Cajio, potrero de Torres, dos leguas, Sur de Guira de Melena, Habana province, Cuba, collected by Felipe Poey (see discussion about type status in Proudlove (2019)).

#### Additional specimens.

ANSP 37111, 70 mm SL, female, Canas, Cuba, collected by C.H. Eigenmann, exact location unknown, 10 March 1903; FMNH 3934, 67 mm SL, male, Canas Cuba, exact location and date unknown; FMNH 33090-91, 67–74 mm SL, females, Cuba, exact location and date unknown; FMNH 52631, 77 mm SL, male, 80 mm SL, female, Cuba, exact location and date unknown; MFP 18.000199, 39.7 mm SL, male, Paredones cave, La Salud locality, Caimito municipality, collected by Erik García-Machado, Pedro Chevalier and Damir Hernández, 18 March 2004; MFP 18.000371 (7 specimens), 41.45–67.5 mm SL, Juanelo Piedra cave, Quibicán municipality, collected by A. Sosa, date unknown; MFP 18.000372, 80.2 mm SL, male, Juanelo Piedra cave, Quibicán municipality, collected by Erik García-Machado, Pedro Chevalier and Damir Hernández, 1 July 2005; MFP 18.000373, 76.2 mm SL female, Juanelo Piedra cave, Quibicán municipality, collected by Erik García-Machado, Pedro Chevalier and Damir Hernández, 1 July 2005; MFP 18.000374, 71.85 mm SL male, Juanelo Piedra cave, Quibicán municipality, collected by Erik García-Machado, Pedro Chevalier and Damir Hernández, 1 July 2005; MFP 18.000198, 74.55 mm SL, male, Juanelo Piedra cave, Quibicán municipality, collected by Erik García-Machado, Pedro Chevalier and Damir Hernández, 1 July 2005; MFP 18.000375 (10 specimens), 27.65–75.6 mm SL, Luis Piedra cave, Quibicán municipality, collected by Alfredo Garcia-Debrás, July 1993; MFP 18.000376 (2 specimens), 52.0–79.4 mm SL, Emilio cave, Ashton formation, Las Cañas locality, Artemisa municipality, collected by Antonio Nuñez Jimenez, 7 November 1943; MFP 18.000377 (4 specimens), 70.5–89.55 mm SL, Emilio cave, Ashton formation, Las Cañas locality, Artemisa municipality, collected by Armando Montoto and Gonzalo Abio, 5 May 1984; MFP 18.000378, 75.0 mm SL, male, Emilio cave, Ashton formation, Las Cañas locality, Artemisa municipality, collected by Erik García-Machado, Pedro Chevalier, Armando Montoto and Lisset Gómez, 25 October 2000; MFP 18.000379, 78.25 mm SL, female, Emilio cave, Ashton formation, Las Cañas locality, Artemisa municipality, collected by Erik García-Machado, Damir Hernández and Didier Casane, 15 December 2008; MFP 18.000380 (3 specimens), 47.3–78.1 mm SL, Baño II cave, Ashton formation, Las Cañas locality, Artemisa municipality, collected by Gonzalo Abio, Erik García-Machado and Armando Montoto, 20 October 1984; MFP 18.000381, 60.25mm SL, female, Baño II cave, Ashton formation, Las Cañas locality, Artemisa municipality, collected by Erik García-Machado, Pedro Chevalier and Damir Hernández, 25 September 2005; MFP 18.000382, 62.3 mm SL, male, Baño II cave, Ashton formation, Las Cañas locality, Artemisa municipality, collected by Erik García-Machado, Pedro Chevalier and Damir Hernández, 25 September 2005; MFP 18.000383 68.7 mm SL, male, Baño II cave, Ashton formation, Las Cañas locality, Artemisa municipality, collected by Erik García-Machado, Pedro Chevalier, Armando Montoto and Lisset Gómez, 25 October 2000; MFP 18.000200, 69.3 mm SL, female, Lechuza cave, Ashton formation, Las Cañas locality, Artemisa municipality, collected by Erik García-Machado, Pedro Chevalier and Damir Hernández, 7 November 2002; MFP 18.000384, 62.0 mm SL, female, El Sitio cave, Ashton formation, Las Cañas locality, Artemisa municipality, collected by José Martínez and Gonzalo Abio, 20 October 1984; MFP 18.000385 (Holotype of *Lucifuga teresinarum*) 71.9 mm SL, male, Lechuza cave, Ashton formation, Las Cañas locality, Artemisa municipality, collected by Erik García-Machado and Armando Montoto, 20 October 1986; MFP 18.000386 (Paratype of *Lucifuga teresinarum*) 78.5 mm SL, male, Baño II cave, Ashton formation, Las Cañas locality, Artemisa municipality, collected by Erik García-Machado and Armando Montoto, 20 October 1986; MFP 18.000387, 77.3 mm SL, female, Baño II cave, Ashton formation, Las Cañas locality, Artemisa municipality, collected by Erik García-Machado, Pedro Chevalier and Damir Hernández, 7 November 2002; MFP 18.000388, 57.3 mm SL, female, Baño II cave, Ashton formation, Las Cañas locality, Artemisa municipality, collected by Erik García-Machado, Pedro Chevalier and Damir Hernández, 7 November 2002; UMMZ 157178 (5 specimens), 52–60 mm SL, Cuba, no further data.

#### Remarks.

Díaz-Pérez (1988) distinguished *L. teresinarum* from *L. subterranea* by the relationships among the dorsal, anal and caudal fins (i.e., independent in the first vs. broadly joined in the second), as well as by the shape of the hood of the male copulatory organ (i.e., broad distal lateral ends in *L. teresinarum* vs. conical in *L. subterranea*); and pointed out that *L. teresinarum* shares both characters states with *L. dentata*. Evidence from molecular data (García-Machado et al. 2011) and morphological considerations indicate that this species is invalid and will be regarded as a synonymy of *L. subterranea*. All measures from the four specimens examined were included within *L. subterranea*. Previous descriptions have indicated that *L. subterranea* has a caudal fin broadly joined to dorsal and anal fins (Poey 1858; Cohen and Robins 1970; Nalbant 1981; Nielsen et al. 1999; Møller et al. 2006). However, as noticed previously by García-Machado et al. (2011) four individuals collected at Baño II cave have the caudal fin joined to the anal but free from the dorsal, a variant previously observed in *L. simile* (Díaz-Pérez et al. 1987a; Díaz-Pérez 1988). The redefinition of *L. teresinarum* as a synonymy of *L. subterranea* increase the morphological variation in this species, only paralleled by *L. simile* (Díaz-Pérez et al. 1987a).

### Lucifuga dentata

Taxon classificationAnimaliaOphidiiformesBythitidae

Poey, 1858

B31359FA-4188-5C84-82C6-84895B9C0393

#### Material examined.

(126 specimens: 63 females, 63 males).

#### Syntypes and/or Poey specimens.

MCZ 12415, 32329, 85–90 mm SL, females, Cave of Cajio, Cuba.

#### Additional specimens.

MFP 18.000312, 93.6 mm SL, female, El Judio cave, Guanahacabibes peninsula, Sandino municipality, collected by José Luis Ponce de León, October 2006; MFP 18.000048, 97.35 mm SL, female, El Judio cave, Guanahacabibes peninsula, Sandino municipality, collected by José Luis Ponce de León, October 2006; MFP 18.000195, 107.1 mm SL, male, El Judio cave, Guanahacabibes peninsula, Sandino municipality, collected by Niurka Hernández, 11 September 2006; MFP 18.000313 100.1 mm SL, female, El Judio cave, Guanahacabibes peninsula, Sandino municipality, collected by José Ponce de León, April 2007; MFP 18.000314 95.1 mm SL, female, El Judio cave, Guanahacabibes peninsula, Sandino municipality, collected by José Ponce de León, April 2007; MFP 18.000315 78.5 mm SL, female, El Judio cave, Guanahacabibes peninsula, Sandino municipality, collected by José Ponce de León, April 2007; MFP 18.000316, 79.95 mm SL, female, El Grillo cave, El Valle locality, Sandino municipality, collected by Yosvani Medina and Damir Hernández, 7 May 2003; MFP 18.000317 (2 specimens), 75.95–100.9 mm SL, La Raja cave, La Jarreta locality, Sandino municipality, collected by Yosvani Medina and Damir Hernández, 6 May 2003; MFP 18.000318 (3 specimens), 93.0–93.5 mm SL, El Jagüey cave, Majin locality, Sandino municipality, collected by Yosvani Medina and Damir Hernández, 30 April 2003; MFP 18.000319 (3 specimens), 75.6–104.3 mm SL, El Patrón cave, Majin locality, Sandino municipality, collected by Yosvani Medina and Damir Hernández, 30 April 2003; MFP 18.000320 (4 specimens), 77.25–97.25 mm SL, Felipe cave, Cayuco locality, Sandino municipality, collected by Erik García-Machado and Pedro Chevalier, 1 March 2001; MFP 18.000321, 81.05 mm SL, female, Pozo Azul sinkhole, Cayuco locality, Sandino municipality, collected by Erik García-Machado, Pedro Chevalier and Damir Hernández, 18 September 2007; MFP 18.000322, 92.5 mm SL, female, Pozo Azul sinkhole, Cayuco locality, Sandino municipality, collected by Erik García-Machado, Pedro Chevalier and Damir Hernández, 18 September 2007; MFP 18.000323, 90.15 mm SL, male, Pozo Azul sinkhole, Cayuco locality, Sandino municipality, collected by Erik García-Machado, Pedro Chevalier and Damir Hernández, 18 September 2007; MFP 18.000324, 82.9 mm SL, female, Pozo Azul sinkhole, Cayuco locality, Sandino municipality, collected by Erik García-Machado, Pedro Chevalier and Damir Hernández, 18 September 2007. South of Havana Province: MFP 18.000325 (2 specimens), 100.0–103.4 mm SL, Paredones cave, La Salud locality, Caimito municipality, collected by Antonio Nuñez Jimenez, date unknown; MFP 18.000326, 105.0 mm SL, female, Paredones cave, La Salud locality, Caimito municipality, collected by José Álvarez Lemus, date unknown; MFP 18.000327, 52.5 mm SL, female, Paredones cave, La Salud locality, Caimito municipality, collected by Erik García-Machado, Pedro Chevalier and Damir Hernández, 18 March 2004; MFP 18.000328, 82.55 mm SL, female, Paredones cave, La Salud locality, Caimito municipality, collected by Erik García-Machado, Pedro Chevalier and Damir Hernández, 18 March 2004; MFP 18.000329, 86.61 mm SL, female, Paredones cave, La Salud locality, Caimito municipality, collected by Erik García-Machado, Pedro Chevalier and Damir Hernández, 18 March 2004; MFP 18.000330, 104.0 mm SL, male, Paredones cave, La Salud locality, Caimito municipality, collected by Erik García-Machado, Pedro Chevalier and Damir Hernández, 18 March 2004; MFP 18.000331, 103.5 mm SL, male, Juanelo Piedra cave, Quibicán municipality, collected by José R. Martínez and Gonzalo Abio, date unknown; MFP 18.000332, 120.0 mm SL, male, Juanelo Piedra cave, Quibicán municipality, collected by Erik García-Machado, Pedro Chevalier and Damir Hernández, 1 July 2005; MFP 18.000333, 102.15 mm SL, male, Juanelo Piedra cave, Quibicán municipality, collected by Erik García-Machado, Pedro Chevalier and Damir Hernández, 1 July 2005; MFP 18.000334, 97.4 mm SL, male, Juanelo Piedra cave, Quibicán municipality, collected by Erik García-Machado, Pedro Chevalier and Damir Hernández, 1 July 2005; MFP 18.000335, 92.5 mm SL, male, Juanelo Piedra cave, Quibicán municipality, collected by Erik García-Machado, Pedro Chevalier and Damir Hernández, 1 July 2005; MFP 18.000336, 90.9 mm SL, female, Juanelo Piedra cave, Quibicán municipality, collected by Erik García-Machado, Pedro Chevalier and Damir Hernández, 1 July 2005; MFP 18.000368 (20 specimens), 75.9–121.2 mm SL, Luis Piedra cave, Quibicán municipality, collected by Alfredo Garcia-Debrás and Abel Ramirez, July 1993; MFP 18.000337 119.0 mm SL, male, Emilio cave, Ashton formation, Las Cañas locality, Artemisa municipality, collected by Erik García-Machado, 20 October 1984; MFP 18.000338, 115.0 mm SL, male, Emilio cave, Ashton formation, Las Cañas locality, Artemisa municipality, collected by Armando Montoto, Javier Vazquez, Erik García-Machado and Pedro A. Díaz, 26 January 1985; MFP 18.000339 (2 specimens), 96.0–99.1 mm SL, Emilio cave, Ashton formation, Las Cañas locality, Artemisa municipality, collected by Armando Montoto and Gonzalo Abio, 5 May 1984; MFP 18.000340 (4 specimens), 84.5–101.9 mm SL, Emilio cave, Ashton formation, Las Cañas locality, Artemisa municipality, collected by Erik García-Machado, Pedro Chevalier, Armando Montoto and Lisset Gómez, 25 October 2000; MFP 18.000342, 86.1 mm SL, male, Baño II cave, Ashton formation, Las Cañas locality, Artemisa municipality, collected by Gonzalo Abio, 20 October 1984; MFP 18.000343 (4 specimens), 67.15–90.25 mm SL, Baño II cave, Ashton formation, Las Cañas locality, Artemisa municipality, collected by Erik García-Machado, Pedro Chevalier, Armando Montoto and Lisset Gómez, 25 October 2000; MFP 18.000196, 82.15 mm SL, female, Baño II cave, Ashton formation, Las Cañas locality, Artemisa municipality, collected by Erik García-Machado, Pedro Chevalier and Damir Hernández, 11 July 2002; MFP 18.000341 (2 specimens), 67.15–90.25 mm SL, Baño II cave, Ashton formation, Las Cañas locality, Artemisa municipality, collected by Erik García-Machado, Pedro Chevalier and Damir Hernández, 11 July 2002; MFP 18.000345 (5 specimens), 74.3–95.25 mm SL, Lechuza cave, Ashton formation, Las Cañas locality, Artemisa municipality, collected by Erik García-Machado, Pedro Chevalier, Armando Montoto and Lisset Gómez, 25 October 2000; MFP 18.000344, 81.0 mm SL, female, El Sitio cave, Ashton formation, Las Cañas locality, Artemisa municipality, collected by José R. Martínez and Gonzalo Abio, 20 October 1984; MFP 18.000346, 85.0 mm SL, male, El Sitio cave, Ashton formation, Las Cañas locality, Artemisa municipality (collection data unknown). South of Matanzas Province: MFP 18.000347, 81.5 mm SL, male, Chicharrones cave, Bolondrón municipality, collected by Erik García-Machado, Pedro Chevalier and Damir Hernández, 28 April 2005; MFP 18.000348, 78.35 mm SL, male, Chicharrones cave, Bolondrón municipality, collected by Erik García-Machado, Pedro Chevalier and Damir Hernández, 28 April 2005; MFP 18.000349, 73.1 mm SL, female, Chicharrones cave, Bolondrón municipality, collected by Erik García-Machado, Pedro Chevalier and Damir Hernández, 28 April 2005; MFP 18.000350, 74.1 mm SL, female, Chicharrones cave, Bolondrón municipality, collected by Erik García-Machado, Pedro Chevalier and Damir Hernández, 28 April 2005; MFP 18.000351, 79.9 mm SL, female, Chicharrones cave, Bolondrón municipality, collected by Erik García-Machado, Pedro Chevalier and Damir Hernández, 28 April 2005; MFP 18.000352, 77.15 mm SL, male, Chicharrones cave, Bolondrón municipality, collected by Erik García-Machado, Pedro Chevalier and Damir Hernández, September 2008; MFP 18.000369 (11 specimens), 69.0–114.15 mm SL, Chicharrones cave, Bolondrón municipality, collected by Alfredo Garcia-Debrás, October 1996; MFP 18.000370 (3 specimens), 74.0–104.0 mm SL, Los Chivos cave, Bolondrón municipality, collected by Alfredo Garcia-Debrás, October 1996; MFP 18.000367 (12 specimens), 65.25–120.1 mm SL, Los Chivos cave, Bolondrón municipality, collected by Alfredo Garcia-Debrás, October 1996; MFP 18.000197, 78.95 mm SL, male, Perico Sánchez cave, Jagüey Grande municipality, collected by Erik García-Machado, Pedro Chevalier and Damir Hernández, 27 April 2005; MFP 18.000353, 69.05 mm SL, female, Perico Sánchez cave, Jagüey Grande municipality, collected by Erik García-Machado, Pedro Chevalier and Damir Hernández, 27 April 2005; MFP 18.000354, 67.45 mm SL, male, Perico Sánchez cave, Jagüey Grande municipality, collected by Erik García-Machado, Pedro Chevalier and Damir Hernández, 27 April 2005; MFP 18.000355, 71.55 mm SL, female, Perico Sánchez cave, Jagüey Grande municipality, collected by Erik García-Machado, Pedro Chevalier and Damir Hernández, 27 April 2005; MFP 18.000356, 72.9 mm SL, female, El Pozo cave, Agramonte municipality, collected by Erik García-Machado, Pedro Chevalier and Damir Hernández, 27 April 2005; MFP 18.000357, 72.95 mm SL, female, El Pozo cave, Agramonte municipality, collected by Erik García-Machado, Pedro Chevalier and Damir Hernández, 27 April 2005; MFP 18.000358, 79.5 mm SL, female, El Pozo cave, Agramonte municipality, collected by Erik García-Machado, Pedro Chevalier and Damir Hernández, 27 April 2005; MFP 18.000359, 66.95 mm SL, female, El Pozo cave, Agramonte municipality, collected by Erik García-Machado, Pedro Chevalier and Damir Hernández, 27 April 2005; MFP 18.000360, 87.05 mm SL, male, El Pozo cave, Agramonte municipality, collected by Erik García-Machado, Pedro Chevalier and Damir Hernández, 27 April 2005; MFP 18.000361, 82.6 mm SL, female, El Pozo cave, Agramonte municipality, collected by Erik García-Machado, Pedro Chevalier and Damir Hernández, 27 April 2005; MFP 18.000362, 114.15 mm SL, male, La Carreta cave, Agramonte municipality, collected by Erik García-Machado, Pedro Chevalier, Didier Casane and Damir Hernández, 26 July 2005; MFP 18.000363, 91.6 mm SL, male, La Carreta cave, Agramonte municipality, collected by Erik García-Machado, Pedro Chevalier, Didier Casane and Damir Hernández, 26 July 2005; MFP 18.000364, 106.9 mm SL, male, La Ratonera cave, Agramonte municipality, collected by Erik García-Machado, Pedro Chevalier, Didier Casane and Damir Hernández, 26 July 2005; MFP 18.000365, 105.2 mm SL, male, La Ratonera cave, Agramonte municipality, collected by Erik García-Machado, Pedro Chevalier, Didier Casane and Damir Hernández, 26 July 2005; MFP 18.000366, 99.2 mm SL, male, La Ratonera cave, Agramonte municipality, collected by Erik García-Machado, Pedro Chevalier, Didier Casane and Damir Hernández, 26 July 2005.

#### Remarks.

*Lucifuga dentata* has been described as having the caudal fin free from dorsal and anal fins and the occiput naked (Poey, 1858; Cohen and Robins, 1970; Vergara, 1980; Nalbant, 1981; Nielsen et al., 1999; Møller et al. 2006). However, we have found that in 60% of the specimens the caudal fin is partially joined to the dorsal and anal fins by tiny basal membranes. Additionally, two individuals, from Luis Piedra caves, have the caudal fin broadly joined to the anal fin. These two conditions were previously assigned as diagnostic for *L. simile* (Díaz et al. 1987a; Díaz 1988). We also found that around 14% of the specimens have the occiput with different degrees of squamation as described for *L. spelaeotes*.

As mention previously for *L. subterranea*, the sampling at localities near to those mentioned in Poey’s original description of the species, applied exactly for *L. dentata*. We also use several exemplars from Juanelo Piedra and Luis Piedra caves which are near to El Cajio cave (ca. 2 km) the type-locality referred by Poey (1858). *Lucifuga dentata* is the most abundant and widely distributed *Lucifuga* species in Cuba. It is found in caves from median-southern karts from central (Matanzas province) to the western part of the island (Guanahacabibes Peninsula). Its distribution is not continuous, with the most important gap between western Havana and Guanahacabibes, Pinar del Río (Hernández et al. 2016).

### Lucifuga simile

Taxon classificationAnimaliaOphidiiformesBythitidae

Nalbant, 1981

C79B8B04-B914-5FAF-84DF-996D8F44F291

#### Material examined.

(22 specimens: 8 females, 14 males).

#### Additional specimens.

MFP 18.000406, (4 specimens), 57.5–100.5 mm SL, Grieta Punta de Guana crevice, Matanzas municipality, North of Matanzas province, Cuba, collected by Gonzalo Abio, Armando Montoto and Erik García-Machado, 6 October 1984; MFP 18.000407, 62.55 mm SL, male, Grieta Punta de Guana crevice, Matanzas municipality, North of Matanzas province, Cuba, collected by Gonzalo Abio, November 1984; MFP 18.000408, 66.65 mm SL, female, Grieta Punta de Guana crevice, Matanzas municipality, North of Matanzas province, Cuba, collected by Alfredo García-Debrás, 8 June 1995; MFP 18.000410, 73.55 mm SL, female, Grieta Punta de Guana crevice, Matanzas municipality, North of Matanzas province, Cuba, collected by Gonzalo Abio, Armando Montoto and Erik García-Machado, 9 September 1984; MFP 18.000409, 84.5 mm SL, female, La Pluma cave, Matanzas municipality, North of Matanzas province, Cuba, collected by Gonzalo Abio, 3 October 1986; MFP 18.000411 (2 specimens), 60.95–92.05 mm SL, La Pluma cave, Matanzas municipality, North of Matanzas province, Cuba, collected by Lazaro Joo, José Alvarez and Ignacio Hernández, 25 March 1984; not catalogued (12 specimens), 66.0–103.0 mm SL, Grieta Punta de Guana crevice, Matanzas municipality, North of Matanzas province, Cuba, (collection data unknown).

#### Remarks.

We examined specimens of *L. simile* from the two known localities: the type-locality Grieta Punta de Guana cave (Nalbant, 1981) and La Pluma Cave (Díaz-Pérez et al. 1987a). This species was also reported from El Tunel cave in Quivican, southern Havana, living in sympatry with *L. dentata* (Díaz-Pérez et al. 1987a). However, this later report need verification.

### 

**Lucifuga lucayana** Møller, Schwarzhans, Iliffe & Nielsen, 2006 

see Møller et al. (2006). 

### 

**Lucifuga spelaeotes** Cohen & Robins, 1970 

see Møller et al. (2006). 

## Supplementary Material

XML Treatment for Lucifuga

XML Treatment for Lucifuga gibarensis

XML Treatment for Lucifuga subterranea

XML Treatment for Lucifuga dentata

XML Treatment for Lucifuga simile
